# The prevalence of the duodenal ulcer promoting gene (*dupA*) in *Helicobacter pylori *isolates varies by ethnic group and is not universally associated with disease development: a case-control study

**DOI:** 10.1186/1757-4749-1-5

**Published:** 2009-03-11

**Authors:** Heather-Marie A Schmidt, Sönke Andres, Nadeem O Kaakoush, Lars Engstrand, Lena Eriksson, Khean-Lee Goh, Kwong Ming Fock, Ida Hilmi, Subbiah Dhamodaran, David Forman, Hazel Mitchell

**Affiliations:** 1 School of Biotechnology and Biomolecular Sciences, University of New South Wales, NSW, Sydney, Australia; 2 Department of Microbiology, Tumor and Cell Biology, Karolinska Institutet, Stockholm, Sweden; 3 Department of Bacteriology, Swedish Institute for Infectious Disease Control, Solna, Sweden; 4 Department of Medicine, Faculty of Medicine, University of Malaya, Kuala Lumpur, Malaysia; 5 Division of Gastroenterology, Department of Medicine, Changi General Hospital, Singapore; 6 Centre for Epidemiology and Biostatistics, Faculty of Medicine and Health, Leeds University, Leeds, UK

## Abstract

**Background:**

The putative *H. pylori *pathogenicity-associated factor *dupA *has been associated with IL-8 induction *in vitro*, and duodenal ulcer (DU) and gastric cancer (GC) development in certain populations, but this association is inconsistent between studies. We aimed to investigate *dupA *prevalence in clinical isolates from Sweden, Australia and from ethnic Chinese, Indians and Malays resident in Malaysia and Singapore and to examine the association with DU and GC. In addition we investigated the sequence diversity between isolates from these diverse groups and compared the level of IL-8 secretion in isolates possessing and lacking *dupA*.

**Methods:**

PCR primers were designed to amplify over the C/T insertion denoting a continuous *dupA*. PCR products from 29 clinical isolates were sequenced and compared with sequences from three additional strains obtained from GenBank. Clinical isolates from 21 Malaysian patients (8 *dupA*-positive, 14 *dupA*-negative) were assessed for their ability to induce IL-8 in AGS cells *in vitro*. Statistical analysis was performed using Fisher's exact test.

**Results:**

The prevalence of *dupA *in isolates from Swedish functional dyspepsia (FD) control patients (65%, 13/20) was higher and in isolates from Indian FD patients (7.1%, 3/42) was lower as compared with isolates from Chinese (28.9%, 13/49, P = 0.005, P = 0.025), Malay (35.7%, 5/14, P = 0.16, P = 0.018) and Australian (37.8%, 17/45, P = 0.060, P < 0.001) FD patients. *dupA *was associated with DU and GC development in Chinese with 62.5% (10/16) and 54.6% (12/22) of isolates possessing *dupA *respectively as compared with FD controls (28.9%) (P = 0.015, P = 0.032). No significant difference in prevalence of *dupA *between FD controls, DU (63.6%, 7/11) and GC (61.9%, 13/21) cases (P = 1.000) was observed in the Swedish population. Sequence analysis revealed a pairwise variation of 1.9% and all isolates possessed the C/T insertion. The average IL-8 induction was 1330 pg/mL for *dupA*-positive isolates and 1378 pg/mL for *dupA*-negative isolates.

**Conclusion:**

Although *dupA *is highly conserved when present, we identified no consistent association between *dupA *and DU or GC development across the ethnic groups investigated, with the *dupA *prevalence in control groups varying significantly. Our results would suggest that in the clinical isolates investigated *dupA *is not associated with IL-8 induction *in vitro*.

## Introduction

Although *Helicobacter pylori *infection invariably results in gastritis, a significant minority of those infected will progress to more severe gastroduodenal pathologies, including Duodenal Ulcer (DU) and Gastric Cancer (GC). The factors resulting in progression to severe *H. pylori-*related disease are poorly understood, however there is compelling evidence to suggest a co-contribution of bacterial virulence factors, host genetics and environmental stimuli [1].

To date a number of *H. pylori *pathogenicity-associated factors, including flagella, adhesins, urease, the vacuolating cytotoxin and the *cag *pathogenicity island (*cag *PAI) have been associated with the development of more serious *H pylori*-related disease outcomes. The *cag *PAI encodes a type IV secretion system (TFSS), homologous to the well-studied *virB/D4 *TFSS of the plant pathogen *Agrobacterium tumefaciens*, as well as the effector protein CagA [2,3]. Translocation of CagA by the TFSS into gastric epithelial cells has been shown to drive deregulation of intracellular signaling pathways resulting in a myriad of cellular effects such as increased motility and elongation [4]. In a process independent of CagA, the TFSS also induces the production of the chemo-attractant and pro-inflammatory cytokine IL-8 by facilitating the entry of peptidoglycan into the host epithelial cell [5]. *In vitro *studies have demonstrated that the *virB4 *homologous ATPase, CagE (*HP0544*, *JHP0492*), is essential for IL-8 induction [6-10]. In addition to *cagE*, multiple *virB4 *homologues exist within the *H. pylori *genome, with both sequenced strains J99 and 26695 possessing three additional *virB4 *homologues [11,12]. Recently Lu *et al. *demonstrated that the partial *virB4 *homologues *JHP0917 *and *JHP0918*, which are present in the plasticity zone of J99 but not 26695, form one continuous gene in clinical isolates the duodenal ulcer promoting gene (*dupA*). and that this gene was a functional *virB4 *homologous ATPase [13]. *dupA*, like *cagE*, is associated with IL-8 induction [13] and thus it is possible that this may be part of an as yet unidentified TFSS. In the original study examining the role of *dupA *in more severe *H. pylori *related disease, *dupA *possessing isolates were found to be associated with the development of DU in patients from Columbia, Japan and Korea [13]. In contrast no association was found with gastric cancer suggesting that *dupA*, and the TFSS it is associated with, may be a major determinant in DU-development. In contrast, two recent studies, one in Brazil [14] and a second in which subjects from Belgium, China, South Africa and the USA were examined [15] did not find an association between *dupA *and DU development. Given that *H. pylori *pathogenicity-associated factors in isolates from different populations have been shown to possess distinct genotypes [16-18], it is important that the association between a newly identified putative pathogenicity-associated factor, such as *dupA *and specific disease outcomes, be assessed in clinical isolates from a range of different regions and ethnic groups [19].

In the present study we determined the prevalence of *dupA *across a range of countries and ethnic groups including Australia, Sweden and the three primary ethnic groups resident within Malaysia, namely Chinese, Indians and Malays and examined the relationship between *dupA *and DU and GC in the Swedish and ethnic Chinese populations. We also sequenced *dupA *PCR products from isolates from each ethnic and disease group and determined that they possessed the C/T insertion required for a continuous *dupA *gene, and additionally that there was a only a small degree of sequence diversity between isolates. Finally, we determined that the induction of IL-8 secretion by AGS cells was not associated with the presence of *dupA *in the clinical isolates investigated *in vitro*.

## Methods

### Patients, *H. pylori *strains and extraction of genomic DNA

#### Malaysian & Singaporean

As part of a larger study examining the role of bacterial, host and environmental factors in GC development, gastric biopsies were obtained from consecutively enrolled unrelated patients undergoing routine endoscopic examination of gastrointestinal symptoms at the University Hospital, Kuala Lumpur, Malaysia and the Changi Hospital, Singapore (between mid-2004 and 2007). Based on endoscopic and histological examination, patients were diagnosed with either distal GC or functional dyspepsia (FD). Gastric biopsies were stored at -70°C in brain heart infusion (BHI) broth containing 20% glycerol and transported to Australia on dry ice. Gastric biopsies were smeared onto campylobacter selective medium (CSA), incubated and identified using morphology, microscopy and biochemical tests as previously described [20,21]. Genomic DNA from one single colony isolate per patient was extracted using the Gentra Puregene DNA extraction kit (Gentra, Minneapolis, Minnesota, USA). In the present study, 142 single colony isolates from 52 Chinese, 42 Indian and 14 Malay FD patients, 22 Chinese GC patients, and 16 Chinese DU patients were included.

#### Swedish

In a previous case-control study which recruited patients from eight hospitals in Sweden biopsies were collected, cultured for *H. pylori *and bacterial DNA extracted as previously described [22]. In the present study 52 single colony isolates from Swedish patients diagnosed with FD (20), DU (11) and GC (21) were included.

#### Australian

In a previous study which recruited patients from Sydney, Australia, biopsies were collected, cultured for *H. pylori *and bacterial DNA extracted as previously described [23]. In the present study, 45 single colony isolates from Caucasian Australian patients with FD were included.

### Primer design and detection of *dupA *in single colony isolates

Primer pair *dupA1274F *and *dupA1674R *(table 1) were designed to amplify a 399–400 bp fragment corresponding to nucleotides 1016315–1016714 in J99 (accession no. AE001439). The PCR primers amplified over the region of *dupA *containing the 1 bp insertion/deletion detected by Lu *et al. *2005 [13], that is the forward primer bound to a sequence from JHP0917 and the reverse primer bound to a sequence from JHP0918, suggesting that PCR positive isolates have both JHP0917 and JHP0918. PCR was performed in a 25 μL reaction containing 0.825 U Taq DNA polymerase (Fisher Biotech, Subiaco, Australia), 45 nmol magnesium-chloride, 10 pmol of each primer and 10 ng of genomic DNA. All PCR runs included a negative control (no DNA) and a positive control (J99). PCR was performed as follows: initial denaturation for 5 minutes at 95°C, followed by 35 cycles of 95°C for 20 seconds, 55°C for 20 seconds and 72°C for 40 seconds, and a final at 72°C for 7 minutes. PCR products were visualized by agarose gel electrophoresis.

**Table 1:** PCR primers used in this study.

**Primer name**	**Nucleotide sequence (5'-3')**	**Reference**
*dupA*113F	GAC GAT TGA GCG ATG GGA ATA T	[15]

*dupA*1083R	CTG AGA AGC CTT ATT ATC TTG TTG G	[15]

*dupA*1274F	GCG TGA TCA ATA TGG ATG CTT TTG C	This study

*dupA*1674R	TTG TCT GGC TCT CAT GTC CGT GTT G	This study

*dupA*1830R	CTT CCT TAT AAG TTT CTT GGT TTG C	[15]

### Validation of primers and investigation of *dupA *by DNA sequencing

29 single colony isolates (4 Chinese, 2 Indian, 4 Malay, 3 Australian and 3 Swedish FD isolates, 4 Chinese and 3 Swedish DU isolates, and 3 Chinese and 3 Swedish GC isolates) were sequenced to validate the specificity of the primers *dupA F *and *dupA *R and to ascertain whether *JHP0917-0918 *formed a single continuous gene in these isolates. Sequencing was performed using the BigDye™ Terminator version 3.1 (Applied Biosystems, Foster City, California, USA). Sequencing analysis was performed on an ABI3730 Capillary DNA sequencer (GENterprise, Mainz, Germany). Sequences were verified using Blast [24] available from Biomanager by ANGIS . Sequence alignments of the 29 strains, with the original *dupA *gene sequence from Gene Bank (accession no. AB196363) [13] and two additional *dupA-*like sequences (accession no. EF076755 and EF076756) available in the GenBank database  were created using ClustalW accurate [25] also available from BioManager.

### Development of a *dupA *multiplex PCR

**Figure 1:**
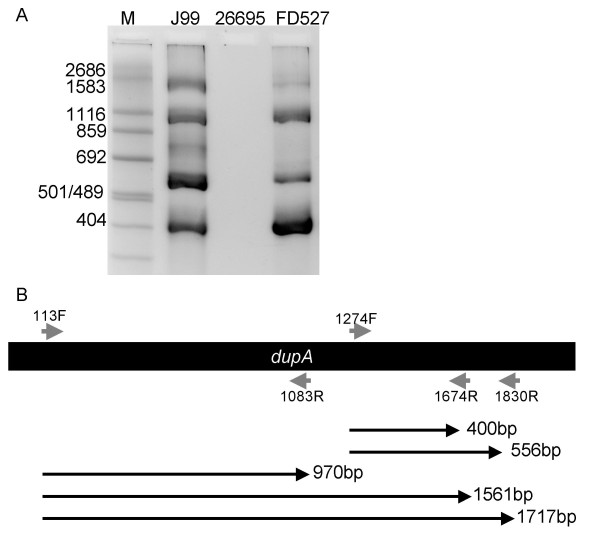
**PCR amplification using the *dupA *multiplex PCR**. (A)Genomic DNA from *H. pylori *strains J99 (positive control), 26695 (negative control) and FD527 (clinical isolate) were used to PCR amplify *dupA *using the forward primers *dupA113*F and *dupA*1274F, and the reverse primers *dupA*1083R, *dupA*1674R and *dupA*1830R. M, size markers (in base pairs). (B) Schematic representation of *dupA *showing the annealing positions of the forward primers *dupA113*F and *dupA*1274F, and the reverse primers *dupA*1083R, *dupA*1674R and *dupA*1830R, and the expected sizes of the amplified PCR products.

To confirm that isolates negative for the *dupA *PCR truly lacked the *dupA *gene, rather than being the result of sequence diversity over the primer annealing site, a multiplex PCR was designed involving the use of 2 forward (*dupA*113F and *dupA*1274F) and 3 reverse (*dupA*1083R, *dupA*1674R and *dupA*1830R) primers which produce 5 differently sized PCR products if all primers bind (Figure 1). Primer sequences are presented in table 1.

The PCR was performed in a 25 uL reaction as described above, with an initial denaturation for 5 minutes at 95°C, followed by 35 cycles of 95°C for 30 seconds, 50°C for 30 seconds and 65°C for 2 minutes, and a final at 65°C for 7 minutes.

### Infection assay and IL-8 induction *in vitro*

AGS cells (ATCC CRL-1739) were routinely maintained in RMPI1640 supplemented with 10% FCS and glutamine (All from Gibco Invitrogen, Paisley, UK), and were cultured at 37°C in 5%CO_2_/95% air. Cells were seeded in 24-well tissue culture plates and infected with approximately 3 × 10^7 ^bacterial cells. 22 *H. pylori *isolates from Malaysian patients (8 *dupA-*positive and 14 *dupA*-negative) were available for *in vitro *analysis. Two *H. pylori *control strains (67:20 (*cag *PAI negative) and 67:21 (*cag *PAI positive) [26] were also available for *in vitro *analysis. *H. pylori *isolates were cultured overnight in brucella broth (Sigma) supplemented with 5% FCS (Gibco Invitrogen). Bacteria were added to AGS cells in duplicate, and were co-incubated for 6 hours before cell culture supernatant was collected and stored at -20°C prior to use. At least three independent experiments were performed; results represent the average for these experiments. The concentration of IL-8 in the supernatant was analysed by ELISA, using the BD OptEIA: Human IL-8 ELISA set (BD, San Diego, CA, USA) according to the manufacturers instructions.

### Statistical Analysis

Fisher's exact test was used to calculate statistical significance; two-sided P values < 0.05 were considered significant.

Sequences have been deposited in GenBank under the following accession numbers: EU253504–EU253532.

## Results

### Prevalence of *dupA*

Following PCR analysis, *dupA *was identified in 15/52 (28.9%) Chinese, 5/14 (35.7%) Malay, 3/42 (7.1%) Indian, 17/45 (37.8%) Australian and 13/20 (65.0%) Swedish FD clinical isolates (table 2). The prevalence of *dupA *in Swedish FD isolates was higher (P = 0.005, P = 0.162, P = 0.025) and the prevalence in Indian FD isolates was lower (P = 0.025, P = 0.0517, P = 0.094) as compared with the Chinese, Malay and Australian FD isolates investigated. There was no significant difference in the prevalence of *dupA *between isolates from Chinese, Malay and Australian FD patients (P = 0.052 to P = 1.000). Among isolates from Chinese or Swedish DU patients there was no significant difference between the two ethnicities, with 10/16 (62.5%) and 7/11 (63.6%) of the isolates possessing *dupA *respectively (P = 1.000). Similarly, there was no significant difference between isolates from Chinese and Swedish GC patients with 12/22 (54.6%) and 13/21 (61.9%) isolates possessing *dupA *respectively (P = 0.759). Among isolates from Chinese patients, the prevalence of isolates possessing *dupA *was significantly higher in isolates from DU and GC patients than in isolates from FD control patients (P = 0.015, P = 0.032), suggesting an association with the development of disease in this population. There was no significant difference in *dupA *prevalence between the isolates from Chinese DU and Chinese GC patients (P = 0.744). Conversely, there was no significant difference in the prevalence of *dupA *between isolates from Swedish FD, DU and GC patients (P = 1.000).

**Table 2:** Comparison of *dupA *prevalence between ethnic groups and countries.

	**Ethnicity/Country**	**Australian**	**Chinese**				**Indian**	**Malay**	**Swedish**		
	
	**Diagnosis**	**FD**	**FD**	**DU**	**GC**	**FD**	**FD**	**FD**	**DU**	**GC**	
Ethnicity	Diagnosis	Prevalence (%)	37.4	28.9	62.5	54.6	7.1	37.7	65.0	63.6	61.9

Australian	FD (n = 45)	37.4	P = 1.000	P = 0.237			P < 0.001	P = 1.000	P = 0.060		

Chinese	FD (n = 52)	28.9	P = 0.237	P = 1.000	P = 0.015	P = 0.032	P = 0.025	P = 0.052	P = 0.005		
	DU (n = 16)	62.5		P = 0.015	P = 1.000	P = 0.744				P = 1.000	
	GC (n = 22)	54.6		P = 0.032	P = 0.744	P = 1.000					P = 0.760

Indian	FD (n = 42)	7.1	P < 0.001	P = 0.025			P = 1.000	P = 0.018	P < 0.001		

Malay	FD (n = 14)	37.7	P = 1.000	P = 0.052			P = 0.018	P = 1.000	P = 0.163		

Swedish	FD (n = 20)	65.0	P = 0.060	P = 0.005			P < 0.001	P = 0.163	P = 1.000	P = 1.000	P = 1.000
	DU (n = 11)	63.6			P = 1.000				P = 1.000	P = 1.000	P = 1.000
	GC (n = 21)	61.9				P = 0.760			P = 1.000	P = 1.000	P = 1.000

Comparison of the prevalence of *dupA*-positive clinical isolates as determined by PCR between FD (functional dyspepsia), DU (duodenal ulcer) and GC (gastric cancer) patients from Australia, Sweden and the 3 primary ethnic groups of Malaysia and Singapore (Chinese, Indian and Malay). The statistical significance (P-values) of each valid comparison is displayed.

### Sequence Analysis

Blast analysis of sequences from 29 clinical isolates revealed significant sequence similarity to the *dupA *gene sequence deposited in GenBank, accession no. AB196363[13] and the sequence of a *dupA*-like protein with GenBank accession no. EF076755. Of the 4 Chinese, 2 Indian, 4 Malay FD, 3 Swedish FD and 3 Australian FD isolates, 4 Chinese and 3 Swedish DU isolates, and 3 Chinese and 3 Swedish GC isolates, all isolates were observed to possess a 1 bp C or T insertion after position 1385 relative to gene *JHP0917 *in J99 suggesting the presence of 1 continuous open-reading frame.

Sequence alignments from the 29 clinical isolates with 3 comparable sequences deposited in GenBank (AB196363, EF076755, EF076756) revealed that *dupA *possessed a pair-wise sequence variation of only 1.90/100 bp (maximum variation: 14.71/100 bp, minimum variation 0.46/100 bp, median variation 0.92/100 bp, standard deviation 3.16/100 bp). Two isolates examined (the Chinese FD isolate FD553 (GenBank sequence: EU253520) and Iranian isolate EF076756), demonstrated a sequence variations of 14.71/100 bp and 12.64/100 bp respectively, being more than 2 standard variations above the mean. In the case of FD553 the high degree of sequence variation was the direct result of a 60 bp insertion at base pair 1465 relative to AB196363. The remaining isolates were within 1 standard deviation of the mean.

### Multiplex PCR

The use of the multiplex PCR revealed no additional isolates positive for *dupA*, when compared to the use of *dupA1274F *and *dupA1674R *alone, suggesting that the use of the single primer pair is adequate to determine the prevalence of *dupA *in both western and east Asian populations. An example of the multiplex PCR is presented in figure 1; all five bands are clearly distinguishable in clinical isolate FD527.

### *In vitro *IL-8 production

**Figure 2:**
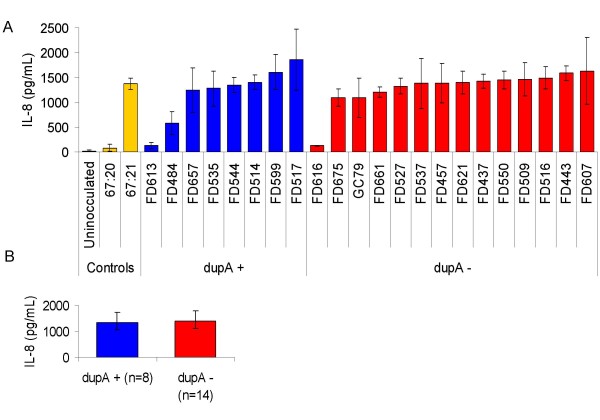
**IL-8 induction in AGS cells by clinical isolates *in vitro***. (A) Induction of IL-8 secretion in AGS cells incubated with indicated strains for 6 hours. The error bars show the standard deviations of each independent experiment. (B) Average induction of IL-8 secretion in AGS cells incubated with *dupA *positive (blue) or negative (red) strains. The error bars show the standard deviations for each group of strains.

The uninoculated control secreted 13 pg/ml, with the negative control strain (67:20) secreting 80 pg/ml and the positive control strain (67:21) secreting 1368 pg/ml (figure 2A). Individual IL-8 secretion levels varied between 126 pg/ml and 1855 pg/ml for *dupA*-positive isolates, and 123 pg/ml and 1633 pg/ml for dupA-negative isolates. However, no significant difference (p > 0.5) was observed in the average level of IL-8 secreted by AGS cells between clinical isolates possessing *dupA *(1330 pg/mL) and those lacking *dupA *(1378 pg/mL) (figure 2B).

## Discussion

The current investigation of the prevalence of *dupA *in clinical isolates from Australia and Sweden as well as from the three major ethnic groups (Chinese, Indians and Malays) resident in Malaysia and Singapore clearly shows that there is significant variability in the prevalence of *dupA *between not only geographical locations, but also between ethnic groups resident in the same country. In the present study the prevalence of *dupA *in *H. pylori *isolates collected from FD patients varied significantly between nationalities/ethnicities, with isolates from Swedish patients being significantly more likely to possess *dupA *(65.0%) than isolates from Australia (26.3%) or ethnic Chinese (28.9%), Indians (7.1%) or Malays (35.7%) resident in Malaysia or Singapore. Interestingly isolates from ethnic Indian FD patients were significantly less likely to possess *dupA *(7.1%) than isolates from any of the other groups. This difference in *dupA *prevalence between countries and ethnic groups is supported by several studies [15,27] including the initial Lu *et al. *2005 [13] which identified that *dupA *was more prevalent in isolates from Columbian gastritis patients (39%) than in isolates from Japanese (14%) and Koreans (7%) gastritis patients. Furthermore, there is no significant difference in *dupA *prevalence reported previously for isolates from Chinese patients (25% and 35.3% respectively) [15,28] and for isolates from ethnic Chinese Malaysians and Singaporeans reported in the present study (28.9%), despite different primer pairs being used, suggesting that the primer pair designed in this study is appropriate. Conversely, the prevalence reported in a north Indian population was considerably higher than that found in the ethnic Indian Malaysians in the present study (16/70 as compared with 3/42) [29]. One possible explanation is that the Indian Malaysians are likely to be predominantly ethnic Tamil [29], originating from Southern India, rather than the north Indian Indeed, two studies in Brazil reported significantly different prevalences for *dupA *(92.3% vs. 62%) [14,30].

We also demonstrated that the association between *dupA *and severe gastroduodenal disease was inconsistent between the Chinese and Swedish populations. In isolates from ethnic Chinese patients the prevalence of *dupA *was significantly higher in patients diagnosed with DU (62.5%) or GC (54.6%) as compared with those diagnosed with FD. This is in contrast to the observations of Lu *et al. *2005 [13] and Zhang *et al*. 2008 [28] who reported a negative association with GC, but supports the observations by Argent *et al. *[15]. In contrast, in the Swedish population there was no significant difference in the prevalence of *dupA *in isolates from patients diagnosed with DU, GC or FD, similar to findings reported from Brazil [14,30]. Our observations are consistent with the reported variation in other *H. pylori *virulence factors such as the *cagA *and *vacA *genes [31], and further emphasise the fact that before a new virulence factor is associated with a specific disease outcome, studies must be undertaken in a range of geographic locations as well in different ethnic groups.

Lu *et al*. have reported that, with the exception of J99, *JHP0917–JHP0918 *forms a continuous open reading frame, *dupA*, due to the presence of a 1 bp C/T insertion in clinical isolates [13]. In the present study, all clinical isolates sequenced possessed a continuous *dupA *gene, signified by the presence of the C/T insertion, a finding that is consistent with previous reports in other populations [13,14,32]. Our results also indicate that *dupA *is highly conserved over the region sequenced with an average partial sequence variation of only 1.9%. This indicates a high degree of conservation comparable to housekeeping genes such as *atpA*, *ureI*, *efp*, and *ppa *[33]. We must acknowledge that as only 29 clinical isolates were sequenced and the sequences used for comparison represent less than 400 bp, the pairwise sequence variation for the entire gene and in a larger population may be higher than that described here. Interestingly in a recent study Douraghi *et al. *compared 3 regions of the *dupA *gene – JHP0917 (289 bp), JHP0918 (259 bp) and the junction region over JHP0917 and JHP0918 i.e. '*dupA' *(216 bp) in 6 Iranian strains with that of 10 Brazilian and 3 Indian strains whose sequences had been deposited in GenBank [32]. The reported sequence similarities in that study ranged from 86.1%–100% for JHP0917, 88–98.8% for JHP0918 and 93.4–99.5% for '*dupA*, a finding that would support the high degree of conservation of this gene observed in our study. Surprisingly in the Douraghi *et al. *study none of the strains were compared for all 3 regions.

The high degree of sequence conservation reported and the finding that *dupA *is present in strains from different continents and in populations with different genetic backgrounds, suggests that *dupA *may confer a fitness advantage of some sort, which has resulted in the conservation of the gene and the sequence, throughout human migration. An alternate explanation, since *dupA *is located in the plasticity zone, is that the acquisition was a relatively recent phenomenon. Although there is, as yet, no specific evidence for either scenario, we believe the former explanation is more likely. Whole gene sequence analysis of *dupA *over an increased sample size is necessary to adequately investigate these points.

Although the initial study by Lu *et al *reported that *dupA *was associated with IL-8 induction *in vitro *in both knockout studies and in clinical isolates [13], in the present study we observed no significant difference in the level of IL-8 induction between strains with or without *dupA*. It is possible however that the strains used in the present study lacked the other essential components of the *dupA *type IV secretion system, however, as none of these components have yet been identified it is impossible to assess whether this is responsible for the lack of association with IL-8 induction in the present study. More comprehensive studies with strains lacking genes known to contribute to IL-8 induction such as *cag *PAI components and with *oipA *off would be beneficial in elucidating the contribution of *dupA *to IL-8 induction.

## Conclusion

Although based on our findings and those of others, it is unlikely that *dupA *is itself a determinant or indicator of a specific clinical outcome across all populations; it may be shown to be an important virulence factor if a role as part of a novel TFSS is identified.

## Competing interests

The authors declare that they have no competing interests.

## Authors' contributions

HMAS designed the study, prepared the Malaysian isolates for analysis, designed and carried out the molecular genetic studies, carried out the sequence alignment and analysis, performed the statistical analysis, helped design and participated in the *in vitro *studies and drafted the manuscript. SA helped design and participated in the *in vitro *studies. NK participated in the molecular genetic studies. LEr prepared the Swedish isolates for analysis. HM was awarded grant to support the study, participated in the design of the study and helped to draft the manuscript. All authors read and approved the final manuscript.
